# Adiponectin in renal fibrosis

**DOI:** 10.18632/aging.102811

**Published:** 2020-02-17

**Authors:** Huan Jing, Simin Tang, Sen Lin, Meijuan Liao, Hongtao Chen, Youling Fan, Jun Zhou

**Affiliations:** 1 The Third Affiliated Hospital of Southern Medical University, Zunyi Medical University, Guangzhou, Guangdong Province, China; 2 The First People’s Hospital of Foshan, Foshan, Guangdong Province, China; 3 Guangzhou Eighth People's Hospital, Guangzhou, Guangdong Province, China; 4 Panyu Central Hospital, Panyu, Guangzhou, Guangdong Province, China

**Keywords:** adiponectin, renal fibrosis

## Abstract

Renal fibrosis is an inevitable consequence of parenchymal scarring and is the common final pathway that mediates almost all progressive renal diseases. Adiponectin, a hormone produced by adipose tissue, possesses potent anti-insulin, anti-inflammatory, and anti-fibrotic properties. Reportedly, adiponectin serves as an important messenger that facilitates complex interactions between adipose tissue and other metabolically related organs. In recent years, a growing body of evidence supports adiponectin involvement in renal fibrosis. These studies provide a deeper understanding of the molecular mechanism of action of adiponectin in renal fibrosis and also offer a potential preventive and therapeutic target for renal fibrosis. In this review, the physiological role of adiponectin is briefly introduced, and then the mechanism of adiponectin-mediated renal fibrosis and the related signaling pathways are described. Finally, we summarize the findings regarding the clinical value of adiponectin in renal fibrotic diseases and prospected its application potential.

## INTRODUCTION

Chronic kidney disease (CKD) is a growing public health issue worldwide with an annual increase in incidence. Renal fibrosis is a well-established final common pathological pathway in almost all cases of chronic kidney injury [1]. Inflammation, oxidative stress, cytokine and signaling cascades, as well as fibroblast proliferation and activation constitute multifactorial etiopathogenetic contributors to renal fibrosis [2–4]. These aforementioned pathophysiological processes lead to hyperadiponectinemia that is associated with renal fibrosis in various presentations of CKD, although its exact mechanism of action remains unclear [5, 6].

Much research has focused on the study of adipose tissue and it is no longer considered an inert fat store of the body. Adipose tissue is now known to be an endocrine organ associated with the synthesis and secretion of several hormones [7]. In this review, we discuss the role of adiponectin (APN), an important adipose tissue-derived hormone. Since it was first discovered in the last century, studies have investigated the biological functions of APN, which is the most abundantly expressed adipokine in organisms. APN mediates many biological processes through its action on specific receptors, including the adiponectin receptor 1 (AdipoR1), adiponectin receptor 2 (AdipoR2), and T-cadherin [8]. Studies have reported that APN participates in the regulation of glycolipid metabolism, increases insulin sensitivity, and possesses anti-inflammatory properties. Much research has focused on the role of APN in delaying renal fibrosis [9].

Herein, we summarize the recent findings regarding the role of adiponectin in renal fibrosis and update the current comprehensive knowledge regarding the usefulness of adiponectin-based treatments in renal fibrosis disease. Additionally, we explored the clinical setting of renal fibrosis to focus on adiponectin as a marker of disease progression or a therapeutic target.

## Adiponectin

### Adiponectin: an overview

APN, an adipocyte-specific plasma protein, consists of 244 amino acid residues and is mainly secreted by white adipose tissue. It is encoded by the ADIOPOQ gene located on chromosome 3q27 [10]. APN exists in the circulation in three forms: a trimer (low molecular weight), a hexamer (medium molecular weight), and high-molecular-weight (HMW) APN [11]. Studies have confirmed high serum APN levels in healthy individuals; APN accounts for approximately 0.01% (5–30 μg/mL) of circulating total protein [12]. Actually, in addition to adipose tissue-derived APN, it is expressed in other tissues, such as human and mouse osteoblasts and liver parenchymal cells [13]. In vivo studies performed by Perri et al. first reported the synthesis and secretion of APN by renal tubular epithelial cells [14]. Serum APN levels are negatively associated with body fat mass, particularly, the visceral fat mass. Research suggests that serum APN is primarily eliminated by the liver and secondarily by the kidneys [13]. Studies have reported that APN monomers (28kDa) and dimers are small enough to cross the glomerular filtration barrier, so these substances may be identified in the urine of healthy individuals. However, HMW APN is also excreted in the urine of patients with proteinuria, which may be secondary to a glomerular filtration barrier imbalance [15].

### Adiponectin receptor

The physiological effects of APN occur secondary to binding of APN to specific receptors on target cell membranes [11]. Currently, the following APN receptors have been identified: AdipoR1, AdipoR2, and T-cadherin [16]. AdipoR1 and AdipoR2 show seven transmembrane domains, and their topology is in contrast to the classical G protein coupled receptor with an intracellular amino terminus and an extracellular carboxy terminus. In contrast, T-cadherin is a unique cadherin molecule that is anchored to the surface membrane by a glycosylphosphatidylinositol moiety and not a transmembrane domain [13]. Numerous studies have confirmed that AdipoR1 is widely distributed throughout the body and also occurs in the kidney, including within the endothelial, mesangial, and proximal tubular cells, as well as podocytes. Additionally, AdipoR2 was observed to be poorly expressed in glomerular and proximal tubular cells [17]. Notably, the adaptor protein, phosphotyrosine interacting with PH domain and leucine zipper 1 (APPL1), one of the APPL isoforms, was the first protein to be identified and interacts directly with APN receptors to mediate APN signaling for enhanced lipid oxidation and glucose uptake [18]. It has been shown that the phosphotyrosine binding domain of APPL1 is directly associated with the intracellular domain of AdipoRs, which regulates both the secretion and metabolic effects of APN [19]. In addition, it was confirmed that APPL1 acts as a protective factor against podocytes injury in high glucose environment [20].

### Role of adiponectin in renal fibrosis

Clinically, conditions such as diabetic nephropathy, immunoglobulin A nephropathy (IgAN), and lupus nephritis (LN), which are associated with CKD can precipitate renal fibrosis [21, 22]. In recent years, a large number of studies have investigated the role of APN in renal fibrosis associated with these diseases, and the consensus based on the available literature confirms the renoprotective effect of APN [23–25]. Clinical and basic research has characterized the significant physiological role of APN, and a strong association has been observed between serum APN levels and outcomes of various renal diseases [26]. Numerous studies have confirmed that decreased APN levels are associated with insulin resistance in patients without CKD. However, in patients with established CKD, APN levels are elevated and disease progression can be positively predicted. Previously, increased APN levels were attributed to reduced APN clearance secondary to renal insufficiency. However, recent studies have confirmed the role of greater expression of APN mRNA, which may also be caused by increased APN secretion. For example, serum APN levels are higher in patients with type 1 diabetes mellitus (T1DM) showing nephropathy [27]. Similarly, compared with healthy controls and non-renal systemic lupus erythematosus (SLE) patients, renally affected SLE patients have higher serum APN levels [28]. Previous studies have suggested that abnormal glycosylation of IgA1 mesangial deposits could significantly contribute to IgAN. Abnormal glycosylation of IgA1 inhibits local APN action with consequent glomerular inflammation and sclerosis that occur in IgAN [29].

### Renoprotective mechanism of adiponectin in renal fibrosis

**Figure 1:**
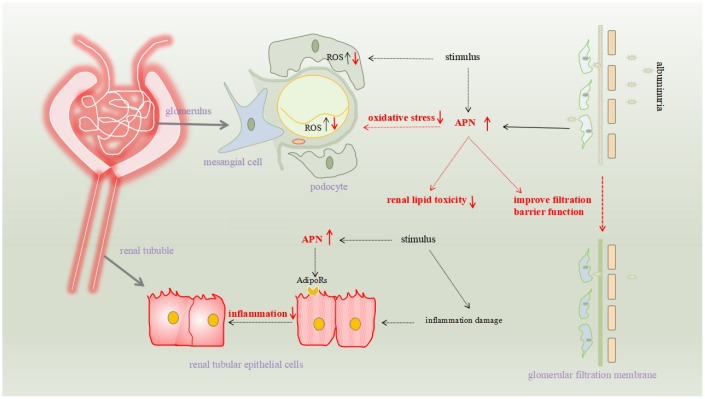
**The pathomechanism of renal fibrosis involves several factors, including oxidative stress and related inflammation, disturbances of glucose metabolism, and hemodynamic abnormalities.** Many studies have confirmed that APN is involved in reducing renal fibrosis, and its specific mechanisms include reducing renal toxicity, reducing renal cell damage, resisting fibrosis, and reducing proteinuria to protect the glomerular filter.

The pathomechanism of renal fibrosis involves several factors, including oxidative stress and related inflammation, disturbances of glucose metabolism, and hemodynamic abnormalities. Many studies have confirmed that APN is involved in reducing renal fibrosis, and its specific mechanisms include reducing renal toxicity, reducing renal cell damage, resisting fibrosis, and reducing proteinuria to protect the glomerular filter (Figure 1).

### Role of adiponectin in reducing renal lipotoxicity

A large body of evidence suggests that disorders of lipid metabolism in the kidney may precipitate kidney injury and dysfunction [30]. APN is known to regulate energy metabolism. AdipoRon, an orally-active synthetic APN agonist, binds to both AdipoRs. Sun Ryoung Choi, et al. demonstrated in vivo experiments in mice that adiponectin can effectively stimulate ceramidase activity associated with its AdipoR1/2 and enhance ceramide catabolism and the formation of its anti-apoptotic metabolite, sphingosine 1 phosphate (S1P). At the same time, they demonstrated that in mouse glomerular endothelial cells (GECs) and podocytes, AdipoRon treatment significantly reduced palmitate-induced lipotoxicity, ultimately improving oxidative stress and apoptosis [31]. Furthermore, based on in vitro experiments, Yaeni Kim et al. reported that AdipoRon ameliorates diabetes-induced renal damage by reducing intrarenal lipotoxicity and oxidative Stress in db/db mice [32].

### Role of adiponectin in reducing renal inherent cell damage

Previous studies have shown that elevated glucose levels increase the mammalian target of rapamycin (mTOR) expression, and overexpression of the mTOR protein can cause kidney injury. Based on in vitro experiments, Sajjad Esmaeili et al. confirmed that APN reduces oxidative stress and associated kidney cell injury by inhibiting mTOR [33]. Notably, oxidative stress is a well-established contributor to renal fibrosis [34]. APN protects against chronic intermittent hypoxia-induced renal cell apoptosis by inhibiting reactive oxygen species (ROS)-induced endoplasmic reticulum stress [35]. Moreover, some studies based on in vivo experiments have confirmed the renoprotective effect of APN. In vivo experiments performed by Fang F et al. confirm that APN-treated mesangial cells can inhibit high glucose-induced ROS production and activation of nicotinamide adenine dinucleotide phosphate oxidase in mice with diabetic nephropathy [36].

### Role of adiponectin in anti-renal fibrosis

The following in vitro studies report that APN may reduce angiotensin II (ANG II)-induced renal fibrosis. Min Tan et al. confirmed that APN inhibits the expression of transforming growth factor beta 1 (TGF- β1) and fibronectin in Ang II- stimulated human renal mesangial cells (HRMCs), thereby reducing the synthesis of extracellular matrix in these cells. This suggests that APN may attenuate Ang II-induced glomerular sclerosis and renal insufficiency [37]. Fei Fang et al. observed that APN inhibits ANG-II-induced nuclear factor kappa light chain enhancer of activated B cell activation and fibronectin expression in proximal tubule cells, thereby attenuating fibrosis [38]. Moreover, many in vivo experiments have confirmed the anti-fibrotic action of APN in the kidney. The mRNA expression and deoxycorticosterone acetate-salt 30 and Ang II infusion-induced production of these fibrotic markers in the renal cortex were reduced in wild type mice showing APN overexpression [25].

### Role of adiponectin in improving the filtration barrier

Injury to the glomerular filtration membrane increases its permeability and affects the barrier function, with consequent renal fibrosis. Endothelial cell and podocyte injury is the chief contributor to glomerular filtration membrane destruction [39, 40]. Proteinuria also contributes to the vicious circle promoting renal fibrosis [41]. Numerous in vivo experiments confirm the importance of APN in maintaining glomerular filtration membrane integrity. Rutkowski et al. developed a mouse model that can induce caspase-8-mediated apoptosis (POD-ATTAC mice), particularly in podocytes. POD-ATTAC mice lacking APN showed significant proteinuria and podocyte ablation. However, POD-ATTAC mice with APN overexpression showed lesser degrees of podocyte injury, improved renal interstitial fibrosis, and partial recovery of renal function. These findings confirm that APN facilitates recovery of injured podocytes and improves renal function [15]. Furthermore, APN overexpression using adenovirus-mediated gene transfer prevents the progression of proteinuria in streptozotocin-induced diabetes in rats. APN reduces proteinuria by increasing renin secretion and improving endothelial dysfunction (by reducing the expression of endothelin 1 and plasminogen activator inhibitor 1 and increasing the expression of renal cortical endothelial nitric oxide synthase) [42]. Research has shown exacerbation of proteinuria and renal fibrosis in a subcutaneous ablation model of APN knockout mice [24]. Moreover, exogenous adiponectin (ADPN) attenuates urinary albumin excretion in diabetic rats and reduces mesangial dilatation and ROS production, thereby preventing interstitial fibrosis [78].

### Signaling pathways

**Figure 2:**
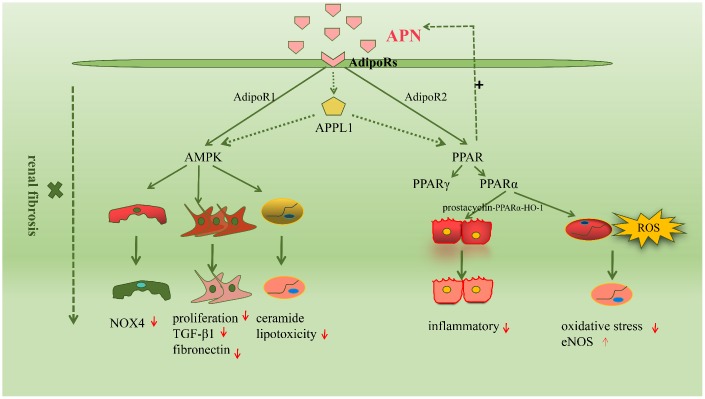
**The molecular mechanisms driving renal fibrosis are wide-ranging and complex, among which signaling pathways are very important.** The common signaling pathways activated in adiponectin-mediated renal fibrosis are the AMPK and peroxisome proliferators-activated receptors (PPARs) pathway.

Renal fibrosis involves a complex interplay between several molecular mechanisms; signaling pathways play an important role in the pathogenesis of this condition [43]. The common signaling pathways activated in APN-mediated renal fibrosis are the adenosine monophosphate-activated protein kinase (AMPK) and peroxisome proliferator-activated receptors (PPARs) pathways. We have summarized these in the following sections. (Figure 2)

### Adenosine monophosphate-activated protein kinase

AMPK is a protein kinase that occurs in most mammalian tissues and acts as an important metabolic switch. Studies have shown that AMPK activation is essential to maintain normal kidney physiology and its function includes activation of the ATP process, inhibition of oxidative stress, and reduction of ROS production [44, 45]. Several studies have confirmed the role of the APN-mediated AMPK signaling pathway in renal fibrosis. Sharma et al. reported that APN prevents or delays the development of albuminuria and maintains the integrity of the foot processes of podocytes, which depends on activation of the AMPK signaling pathway in podocytes [46]. Fang et al. reported that the APN signaling pathway regulates oxidative stress, podocyte foot fusion and the onset of proteinuria through in vivo and in vitro actions of AMPK [38]. These authors also observed that APN controls oxidative stress and glomerular cell division through its action on the glomerular filtration barrier and AdipoR1-mediated activation of AMPK [47]. Similarly, APN transport across the glomerular filtration barrier may inhibit renal inflammation and oxidative stress by activating AMPK-activated protein kinase and preventing proteinuria in vivo [48]. Recent studies have proved that the AMPK pathway plays an important role in the inhibitory action of APN on Ang II-induced synthesis of TGF-β1 and fibronectin in HRMCs [37]. Moreover, studies have shown that APN activates the AMPK signaling pathway through AdipoRs to protect the kidneys in patients with diabetic nephropathy [31]. APN can also alleviate mitochondrial cytopathy-induced renal dysfunction through the AMPK-mTOR signaling pathway [49]. In conclusion, several studies have confirmed the role of AMPK-related signaling pathways in APN-mediated renal fibrosis; however, many questions remain unanswered in this regard.

### Peroxisome proliferator-activated receptors

PPARs are members of the nuclear receptor superfamily, mainly comprising PPAR-γ and PPAR-α, which regulate several biological functions, such as cell growth, differentiation, and apoptosis [50]. PPARs directly and indirectly protect against renal injury [51, 52]. Several studies have confirmed that increased APN expression may be attributed to PPAR-γ activation, because APN agonists are known to regulate several adipocytokines in adipose tissue and upregulate APN transcription [53]. Moreover, the renoprotective effect of APN is attributed to reduced oxidative stress and AdipoR2-induced activation of the PPAR-α signaling pathway [54]. Previous studies have reported that the APN-mediated prostacyclin-PPARα-heme oxygenase-1 signaling pathway protects against renal ischemia/ reperfusion injury [55]. Furthermore, it has recently been shown that γ-oryzanol improves obesity-induced nephropathy by regulating the AdipoR2/PPAR-α axis [56]. Recent evidence has shown that AdipoR2-induced activation of PPARα upregulates the genes involved in fatty acid transport and binding and also activates peroxisomal and mitochondrial fatty acid β-oxidation, thereby promoting fatty acid catabolism. PPARα-mediated gene transcription upregulates the PPAR gamma coactivator-1 alpha and estrogen-related receptor alpha axes, enhances mitochondrial oxidative capacity, reduces oxidative stress, and also reduces lipid accumulation in target cells in the kidney [15].

### Other signaling pathways

In addition to the therefore mentioned signaling pathways, adiponectin may also mediate others signaling pathways. However, there is limited evidence in this regard. Recent studies have reported that APN triggers the mTOR/p mTOR/SIRT1 signaling pathway to prevent cell death in the human kidney [57]. Additionally, the renoprotective action of APN is attributed to increased expression of bone morphogenetic protein-7 and Smad-7 and reduced expression of TGF-β1 and Smad-3 to maintain the balance of the TGF-β1/Smad signaling pathway.

## Potential clinical value of adiponectin in renal fibrosis

Clinical and basic studies have confirmed the renoprotective action of APN in patients with renal fibrosis. Therefore, attention is presently being focused on the clinical value of APN in the treatment of renal diseases. The current research mainly focuses on two aspects: The first is that changes in plasma or urine APN can be used as biomarkers of renal injury levels (Table 1). Many researchers believe that plasma APN concentrations increase with the progression of renal insufficiency, possibly due to positive feedback regulation to renal damage. In addition, studies have suggested that CKD patients do not have the beneficial effects of high APN levels, probably because these patients have higher circulating inflammatory cytokines, greater insulin resistance, and accelerated atherosclerosis [58]. There are also some other opinions that in the environment of end-stage renal disease, APN receptor signaling is blocked after AMPK phosphorylation, thereby reducing the beneficial effects of APN [59]. The second is the treatment strategy for APN, including increasing APN levels and APN receptor levels or through APN receptor agonists.

**Table 1:** Significance of APN as biomarkers for various renal diseases.

**Renal diseases**	**Biomarkers**	**Significance**
**T1DN**	Secrum APN	Predict mortality; Predict progression from macroalbuminuria to ERSD
	Urinary APN	Predict the decline of renal function
**T2DN**	Urinary HMW-APN excretion	Early prediction of renal failure
	Secrum APN	Predict proteinuria
**LN**	Secrum APN	Predict proteinuria
	Urinary APN	Increase in SLE patients with renal involvement
**IgAN**	Secrum APN	Predict atherosclerosis

### Adiponectin as a potential biomarker in renal fibrosis

### Diabetic nephropathy

Diabetic nephropathy is the most common and serious etiopathogenetic contributor to end-stage renal disease (ESRD) [60]. Several studies have reported that APN is a useful predictive biomarker of diabetic nephropathy [61–63]. Studies have reported elevated serum APN levels in patients with T1DM and T2DM who show nephropathy and also that serum APN levels are independent predictors of progressive diabetic kidney disease (DKD) [54]. Mayumi Yamamoto et al. confirmed elevated urinary APN in patients with declining renal function and that this parameter could be used as an alternative biomarker of diabetic nephropathy [64]. APN has also been investigated as a biomarker for the progression of diabetic nephropathy in T1DM. A previous study suggested that high serum APN levels could predict mortality and progression to ESRD in patients with T1DM [65]. Additionally, some studies suggest that urinary APN is a powerful independent predictor of progression from macroalbuminuria to ESRD, which has a significant predictive effect on biomarkers in patients with type 1 diabetic nephropathy [66, 67]. It is noteworthy that the increase in serum total APN in patients with T1DM showing nephropathy is secondary to the increase in HMW APN [68]. Urinary HMW APN excretion is an independent predictor of renal dysfunction in patients with T2DM with early DKD [37]. Recently, ARLR, A et al. reported that serum APN levels can predict proteinuria in patients with T2DM, and compared with patients showing proteinuria, patients with T2DM and microalbuminuria showed significantly higher serum APN levels [69].

### Lupus nephritis

LN is the most serious form of SLE, which is also one of the most common causes of renal fibrosis [70]. Rovin et al. reported that compared with healthy controls and patients with nonrenal SLE, those with renal SLE show higher serum APN levels. However, multivariate analysis did not confirm this finding. A significant association between serum APN levels and renal involvement was observed only on univariate analysis, and this association was nonsignificant after adjusting for confounders. Therefore, the significance of high serum lipid levels in SLE and the association between these high serum lipids and renal involvement remains unclear. Notably, urinary APN is a relatively sensitive marker of renal SLE flares [71]. Maryam et al. reported that urinary APN levels increased significantly in patients with SLE showing renal involvement. APN levels can be used as a biomarker to differentiate between patients with SLE presenting with and without renal impairment [72]. A recent study reported that high serum APN levels are associated with proteinuria in patients with LN. Multivariate analysis performed after adjusting for confounders showed that elevated serum APN levels continued to show a significant association with the severity of proteinuria. Therefore, a high serum APN level can be considered a useful biomarker of proteinuria in patients with LN [73].

### IgA nephropathy

IgAN is a common condition associated with renal fibrosis; however, there is limited evidence to conclusively prove the association between APN and IgAN. Studies have confirmed increased urinary APN excretion in patients with IgAN showing massive proteinuria [74]. Furthermore, it has been suggested that the serum total APN level is an independent predictor of atherosclerosis in patients with IgAN [75]. Despite the limited evidence in the currently available literature, there is much scope for future research to conclusively establish serum APN as a biomarker of renal fibrosis in IgAN.

### Adiponectin-associated renal fibrosis treatment

**Figure 3:**
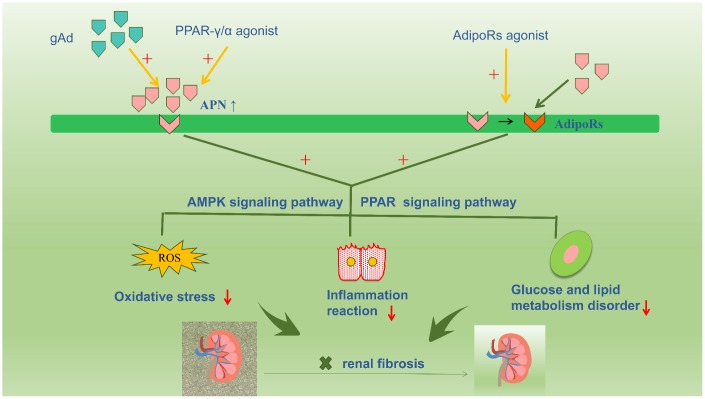
**Accumulative evidence about the renoprotective role of adiponectin promotes a therapeutic strategy for renal fibrosis targeting adiponectin, such as increasing the plasma adiponectin level or increasing the sensitivity of adiponectin by activating adiponectin receptors.** Numerous studies have confirmed that PPAR-γ agonist compounds increase adiponectin secretion and circulating adiponectin levels in adipose tissue. And the exogenous adiponectin is also currently a hot focus for the treatment of kidney disease. In addition, AdipoRon directly activates intrarenal AdipoR1 and AdipoR2, and promotes downstream reactions, thereby restrainting renal fibrosis.

Accumulated evidence in the available literature highlights the renoprotective action of APN, which is increasingly being viewed as a novel therapeutic target for renal fibrosis via interventions to increase serum APN levels or enhance APN sensitivity by activating APN receptors (Figure 3). Numerous studies have confirmed that PPAR-γ agonists increase APN secretion, thereby increasing circulating APN levels in adipose tissue [76, 77]. Notably, unlike PPAR ligands, APN does not cause volume overload. Therefore, exogenous APN could be a useful therapeutic agent in patients with diabetic nephropathy. However, further clinical interventional trials based on stringent criteria are warranted to definitively establish the role of APN [78]. Basic studies show that PPARα/γ dual receptor agonists (tesaglitazar) can increase serum APN levels to suppress T2DM [79]. Resveratrol prevents diabetic nephropathy via the following mechanism: resveratrol activates the AMPK-SIRT1-PGC-1alpha axis and PPARα by increasing AdipoR1 and AdipoR2 expression and prevents high glucose-induced oxidative stress and cell apoptosis [80]. Additionally, AdipoRon directly activates intrarenal AdipoR1 and AdipoR2 and promotes downstream reactions, thereby restrainting renal fibrosis; this effect is unrelated to the systemic effect of APN. Studies have reported that AdipoRon may be a promising agent to treat diabetic nephropathy by stimulating intracellular calcium ions, activating the AMPK-LKB1/PPARα signaling pathway, and increasing ceramidase activity via activation of AdipoRs and downstream targets [32].

## Special: Adiponectin promotes renal fibrosis

Although most current studies report the renoprotective effect of APN, a few authors have expressed differing opinions. Jun Yang et al. describe a novel mechanism by which APN mediates the pathogenesis of renal fibrosis. In response to renal tissue injury, APNincreases Th2 cytokine production and monocyte-to-fibroblast transition, which contribute significantly to the pathogenesis of renal interstitial fibrosis. These data suggest that inhibition of APN/AMPK signaling mayserve as a novel therapeutic approach in patients with CKD [81]. APN secreted in specific environments (such as following lipopolysaccharide exposure) has also been shown to promote renal fibrosis [82]. Studies have shown that prevention of Klotho downregulation protects against kidney injury, delays CKD, and improves kidney function [83]. APN reduces Klotho secretion in renal tubular epithelial cells, which indirectly implicates APN in the causation of kidney injury [84]. Further research is warranted to conclusively establish the complex role of APN in renal fibrosis.

## CONCLUSION

APN (a type of adipocytokine) plays a key role in the onset and progression of renal fibrosis in several diseases. Overwhelming evidence suggests the renoprotective actions of APN, which binds the APN-related receptors, triggers several signaling pathways, reduces oxidative stress and inflammation, regulates lipid and glucose metabolism, and consequently reduces renal fibrosis. However, a few clinical studies have reported that high serum APN levels are associated with hyperalbuminuria and worsened renal function. This finding could be attributed to a compensatory endogenous effort of the body to salvage the injured kidney by producing more protective factors. Currently, APN has attracted much attention in this regard; however, its exact role in renal fibrosis remains unclear. Further clinical studies are warranted to translate the experimental evidence regarding the renoprotective role of APN into real-world clinical practice to offer newer therapeutic targets for renal fibrosis.
